# Feasibility of endoscopic evaluation of *Helicobacter pylori* infection status by using the Kyoto classification of gastritis in the population‐based gastric cancer screening program: A prospective cohort study

**DOI:** 10.1002/hsr2.325

**Published:** 2021-07-14

**Authors:** Ryosuke Hirai, Mami Hirai, Yuichi Shimodate, Mariko Minami, Sho Ishikawa, Takafumi Kanadani, Rio Takezawa, Akira Doi, Naoyuki Nishimura, Hirokazu Mouri, Kazuhiro Matsueda, Hiroshi Yamamoto, Motowo Mizuno

**Affiliations:** ^1^ Department of Gastroenterology and Hepatology Kurashiki Central Hospital Okayama Japan; ^2^ Department of Gastroenterology and Hepatology Okayama University Hospital Okayama Japan

**Keywords:** atrophic gastritis, cancer screening, gastric cancer, gastrointestinal endoscopy, *Helicobacter pylori*

## Abstract

**Background and aims:**

We have started a new population‐based endoscopic gastric cancer screening program in Kurashiki city with consideration of *Helicobacter pylori* infection status based on endoscopic features. We aimed to verify the feasibility of this attempt in a prospective case‐registration study (UMIN000028629).

**Methods:**

Data were collected from 1784 subjects without past eradication of *H. pylori* and who underwent endoscopic gastric cancer screening in Kurashiki Central Hospital Preventive Healthcare Plaza from September 2017 to June 2018. Endoscopic judgment of *H. pylori* infection status was made according to the Kyoto classification of gastritis. For comparison, a combination serum test of anti‐*H. pylori* antibody and pepsinogen I and II, the ABC method, was used.

**Results:**

The endoscopic diagnoses were nongastritis, 1215 (68.1%); active or inactive gastritis, 469 (26.3%); and undefined, 23 (1.3%). With the ABC method as a reference standard, the false‐negative rate of the endoscopic judgment for *H. pylori* infection was 16.3% (95% confidence interval: 13.1%‐20.0%). Most false‐negative cases were of Group B in the ABC method, which is considered gastritis with mild mucosal atrophy. Antibody titers in this population were mostly in the weak‐positive range but clinically significant elevation of the antibody suggesting current infection was observed in some cases.

**Conclusions:**

Endoscopic diagnosis of *H. pylori* infection status in a population‐based gastric cancer screening program is mostly reliable, but false‐negative results may occur, especially in patients with mild gastric atrophy. To avoid this limitation, we recommend adding *H. pylori* antibody test to the program.

## 1 INTRODUCTION

Gastric cancer is decreasing in incidence, but it is still one of the major causes of cancer death in Japan. *Helicobacter pylori* is believed to be the pathogen responsible for developing gastric cancer.^1^, ^2^ Eradication of *H. pylori* reduces the gastric cancer risk^3^, ^4^, ^5^, ^6^, ^7^ and mortality,^8^ but the risk still remains even in the second decade after eradication.^9^ The persuasive evidence of *H. pylori* in causation of gastric cancer notwithstanding, the population‐based gastric cancer screening program in Japan has operated without considering patients' *H. pylori* infection status, that is, radiographic screening every year for everyone above 40 years of age. In 2015, endoscopic screening was reported to reduce gastric cancer mortality by 67% compared with radiographic screening,^10^ and esophagogastroduodenoscopy (EGD) has become an option for population‐based gastric cancer screening programs since 2016. However, gastric cancer is still being surveilled without taking *H. pylori* infection into account, that is, EGD every 2 years in all people above 50 years of age; any tests for *H. pylori* infection, including serum anti‐*H. pylori* antibody measurement, are not supported financially in the government‐directed screening program.

In 2014, the Kyoto classification of gastritis was announced, and evaluating the status of *H. pylori* infection according to endoscopic findings of gastritis has been found reliable.^11^, ^12^ Thus, in 2017, we began our population‐based endoscopic gastric cancer screening program in Kurashiki city, in which evaluation of *H. pylori* infection according to the Kyoto classification of gastritis was conducted as part. If this attempt works, gastric cancer screening with consideration of *H. pylori* infection can be achieved without further expense or change of the program. A major concern about this approach was that endoscopists in the screening program had various levels of skills and knowledge for judging endoscopic findings of gastritis according to the Kyoto classification.

Another method with links to gastric atrophy and *H. pylori* infection status in assessment of gastric cancer risk is the ABC method. It is a combination of a serum test for *H. pylori* antibody and serum concentrations of pepsinogen (PG). In subjects with severe atrophic gastritis after long‐time *H. pylori* infection, checking *H. pylori* antibody titers alone is often insufficient to detect past, or even current, *H. pylori* infection, which can be detected by adding the PG test, a marker for gastric mucosal atrophy.^13^ The ABC method has been touted as an effective nonendoscopic mass‐screening method for gastric cancer^14^ and was shown useful also for evaluation of *H. pylori* infection status,^15^ but the method is not supported in the population‐based screening program by the government.

In this work, we sought to determine if we could reliably diagnose *H. pylori* infection status using the Kyoto classification of gastritis as part of our population‐based gastric cancer screening program. To assess this possibility, we conducted a prospective case‐registration cohort study and compared the results of *H. pylori* infection status judged endoscopically according to the Kyoto classification of gastritis with results of the serum ABC method as a reference standard.

## 2 MATERIALS AND METHODS

### 2.1 Subjects

Subjects were those over 20 years of age who underwent EGD for gastric cancer screening in Kurashiki Central Hospital Preventive Healthcare Plaza affiliated to Kurashiki Central Hospital from September 2017 to June 2018 and agreed to participate in this study. All subjects underwent blood tests of the ABC method and EGD on the same day. Information collected on each subject was age, sex, family history of gastric cancer, history of examination for or eradication of *H. pylori*, use of acid‐secretion inhibitor, and history of gastric or abdominal surgery. Subjects who were pregnant, had a history of gastric surgery, or had eradication therapy of *H. pylori* were excluded. We planned to enroll arbitrarily around 2000 subjects by considering the annual number of recipients of endoscopic gastric cancer screening at our facility, the study period, and the range of 95% confidence interval (CI) of the results.

The study was conducted according to the guidelines of the Declaration of Helsinki. The study was approved by the institutional review board of the Kurashiki Central Hospital and was registered with UMIN Clinical Trials Registry (UMIN000028629). The objective of the study was explained to all subjects before their participation, and a written informed consent was obtained from each subject.

### 2.2 Endoscopic evaluation of *H. pylori* infection according to the Kyoto classification of gastritis

EGD was performed with a scope of EG‐L580NW and a light source device of LASEREO (FUJIFILM Medical Co., Ltd., Tokyo, Japan). At endoscopic examination, we evaluated and recorded the following endoscopic findings together with the gastric cancer screening: grade of atrophy according to the endoscopic atrophy scale described by Kimura and Takemoto^16^, ^17^ and items described in the Kyoto classification of gastritis, that is, presence or absence of regularly arranged collecting venules, enlarged gastric folds, nodularity, diffuse redness, spotty redness, map‐like or patchy redness of the gastric mucosa, xanthoma, and/or foveolar‐hyperplastic or fundic grand polyp. On the basis of these findings, gastritis and evaluation of the status of *H. pylori* infection were classified as nongastritis (never infected with *H. pylori*); active gastritis (current *H. pylori* infection); inactive gastritis (past infection)^11^; or undefined (equivocal or status of gastritis difficult to judge). The main diagnostic criteria were gastric mucosal atrophy with diffuse and/or spotty redness for active gastritis; atrophy with map‐like redness and/or patchy redness for inactive gastritis; and regular arranged collecting venules in the lesser curvature of the gastric angle for nongastritis. Endoscopists in the study were resident doctors as well as senior, experienced doctors of our department; in cases where EGD was performed by noncertified endoscopists, the endoscopic findings were checked by board‐certified endoscopists of the Japan Gastroenterological Endoscopy Society or the Japanese Society of Gastroenterology.

### 2.3 Serum test for *H. pylori* infection

For serum diagnosis of *H. pylori* infection, we used the ABC method^14^: Serum antibody to *H. pylori* was measured by an enzyme immunoassay method with E‐plate (Eiken Chemical, Tokyo, Japan); a cutoff value of ≥3 U/mL was used, which is a value recommended for the gastric cancer screening program to reduce the frequency of false‐negative results^18^, ^19^ (an original cutoff value for clinical practice was ≥10 U/mL^14^). PGI and II were measured by the chemiluminescent enzyme immunoassay method, Fujirebio, Tokyo, Japan); results were considered positive for gastric atrophy when PGI and PG I/II ratios were ≤70 ng/mL and ≤3.0, respectively.^20^ The combined results of the antibody and PG values were classified as Group A, *H. pylori* antibody (−) PG (−); Group B, *H. pylori* antibody (+) PG (−); group C, *H. pylori* antibody (+) PG (+); and group D, *H. pylori* antibody (−) PG (+)^14^; the risk of gastric cancer is thought to be progressively higher from A to D. In terms of *H. pylori* infection status and grade of gastric atrophy, Group A was thought to be never infected with *H. pylori* and to have no gastric atrophy; Group B, current or past *H. pylori* infection with mild gastric atrophy; group C, current or past infection with advanced gastric atrophy; and group D, past infection with severely advanced atrophy.^14^

### 2.4 Outcomes

The primary outcomes were the false‐negative rate for detection of *H. pylori* infection, either current or past, of the endoscopic evaluation according to the Kyoto classification of gastritis compared with the results of the ABC methods and the false‐negative rate of the ABC method compared with results of the endoscopic evaluation. The secondary outcome was factors associated with the false‐negative results of the endoscopic judgment for *H. pylori* infection.

### 2.5 Statistical analysis

We calculated 95% CIs of the false‐negative or false‐positive rates using the Clopper‐Pearson method. To assess the factors associated with the false‐negative results of the endoscopic judgment, we used logistic regression analysis including factors which were likely to affect endoscopic findings. Odds ratios and 95% CIs were calculated, and *P* < .05 was considered statistically significant. For the statistical analysis, we used EZR (Saitama Medical Center, Jichi Medical University, Saitama, Japan), which is a graphical user interface for R (the R Foundation for Statistical Computing, Vienna, Austria).

## 3 RESULTS

**FIGURE 1:**
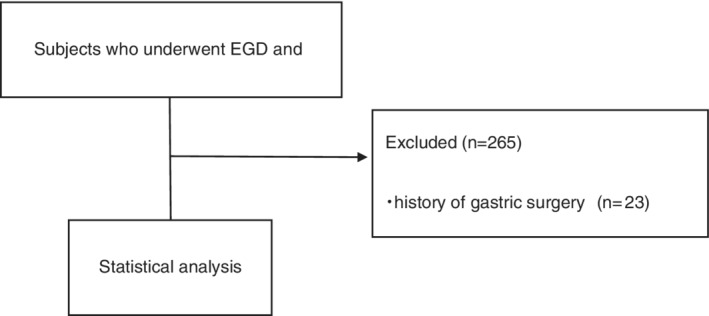
Patients flow of the study

During September 2017 to June 2018, 2049 subjects were approved to participate in the study. Among them, 23 with history of gastric surgery and 242 with posteradication of *H. pylori* were excluded; 1784 subjects were enrolled (Figure 1). Characteristics of the study subjects are presented in Table 1. The median age was 54 years, and 51.3% were male. Gastric cancer was detected in seven subjects (0.4%) and adenoma in two subjects (0.1%). The results of the ABC method were Group A, n = 1312 (73.5%) and Group B ~ D, n = 472 (B, 335 (18.8%); C, 121 (6.8%); and D, 16 (0.9%)); characteristics by the ABC groups are provided in Table S1. The prevalence of current or past *H. pylori* infection according to the ABC methods was 26.5% in the study subjects.

**TABLE 1:** Characteristics of study subjects (n = 1784)

Age (years)	54.0 (±11.8)
Male	915 (51.3%)
Medication	180 (10.1%)
Histamine H2–receptor antagonist	21 (1.2%)
Proton pump inhibitor	83 (4.7%)
Potassium‐competitive acid blocker	10 (0.6%)
Gastric neoplasms	
Cancer	7 (0.4%)
Adenoma	2 (0.1%)
Kyoto classification of gastritis	
Atrophy	469 (26.3%)
Intestinal metaplasia	211 (11.8%)
Diffuse and/or spotty redness	599 (33.6%)
Mucosal swelling and/or enlarged fold	445 (24.9%)
Nodularity	21 (1.2%)
RAC on angular region or antrum	1320 (74.0%)
ABC method^a^	
A	1312 (73.5%)
B	335 (18.8%)
C	121 (6.8%)
D	16 (0.9%)

^a^ The ABC method: Group A, *Helicobacter pylori* antibody (−) PG (−); Group B, *H. pylori* antibody (+) PG (−); group C, *H. pylori* antibody (+) PG (+); and group D, *H. pylori* antibody (−) PG (+). PG, pepsinogen; positive if PGI and PG I/II ratios were ≤70 ng/mL and ≤ 3.0, respectively.

Abbreviation: RAC, regular arrangement of collecting venules.

The endoscopic diagnoses of gastritis according to the Kyoto classification were nongastritis, 1292 (72.4%); active or inactive gastritis, that is, current or past *H. pylori* infection, 469 (26.3%); and undefined, 23 (1.3%) (Table 2). With the ABC methods as a reference standard for *H. pylori* infection, the false‐negative rate of the endoscopic judgment for *H. pylori* infection was 16.3% (77/472, 95% CI: 13.1%‐20.0%), and the false‐positive rate was 6.8% (89/1312, 95% CI: 5.5%‐8.3%). The false‐negative rate of the ABC method, with endoscopic diagnosis of gastritis as a reference, was 19.0% (89/469, 95% CI: 15.5%‐22.8%), and the false‐positive rate was 6.0% (77/1292, 95% CI: 4.7%‐7.4%).

**TABLE 2:** Relationship between the diagnosis of *Helicobacter pylori* infection status according to the Kyoto classification of gastritis and the ABC method

Endoscopic diagnosis	ABC method	
	Group A 1312	Group B‐D 472
Nongastritis (1292, 72.4%)	1215	77^a^
Active or inactive gastritis (469, 26.3%)	89^b^	380
Undefined (23, 1.3%)	8	15

^a^ With the ABC methods as a reference standard for *Helicobacter pylori* infection, the false‐negative rate of the endoscopic judgment for *H. pylori* infection was 16.3% (77/472, 95% CI: 13.1%‐20.0%).

^b^ The false‐negative rate of the ABC methods, with endoscopic judgment of *H. pylori* status as a reference, was 19.0% (89/469, 15.5%‐22.8%).

In cases with discrepancy between the endoscopic judgment and the ABC method, the recorded endoscopic images were carefully re‐examined by two experienced endoscopists (MM, RH). The endoscopic diagnosis of gastritis after the re‐evaluation was nongastritis in 1315 (73.7%) and active or inactive gastritis in 446 (25.0%) (Table 3). After the re‐evaluation, the false‐negative rate of the endoscopic judgment for *H. pylori* infection decreased to 11.0% (52/472, 95% CI: 8.3%‐14.2%), and the false‐negative rate of the ABC method decreased to 9.2% (41/446, 95% CI: 6.7%‐12.3%).

**TABLE 3:** Relationship between the diagnosis of *Helicobacter pylori* infection status according to the Kyoto classification of gastritis and the ABC method after re‐evaluation of endoscopic findings by board‐certified experienced endoscopists

Endoscopic diagnosis	ABC method	
	Group A 1312	Group B‐D 472
Nongastritis (1315, 73.7%)	1263	52^a^
Active or inactive gastritis (446, 25.0%)	41^b^	405
Undefined (23, 1.3%)	8	15

^a^ The false‐negative rate of the endoscopic judgment for *H. pylori* infection after the re‐evaluation of endoscopic findings was 11.0% (52/472, 95% CI: 8.3%‐14.2%).

^b^ The false‐negative rate of the ABC methods was 9.2% (41/446, 6.7%‐12.3%).

**FIGURE 2:**
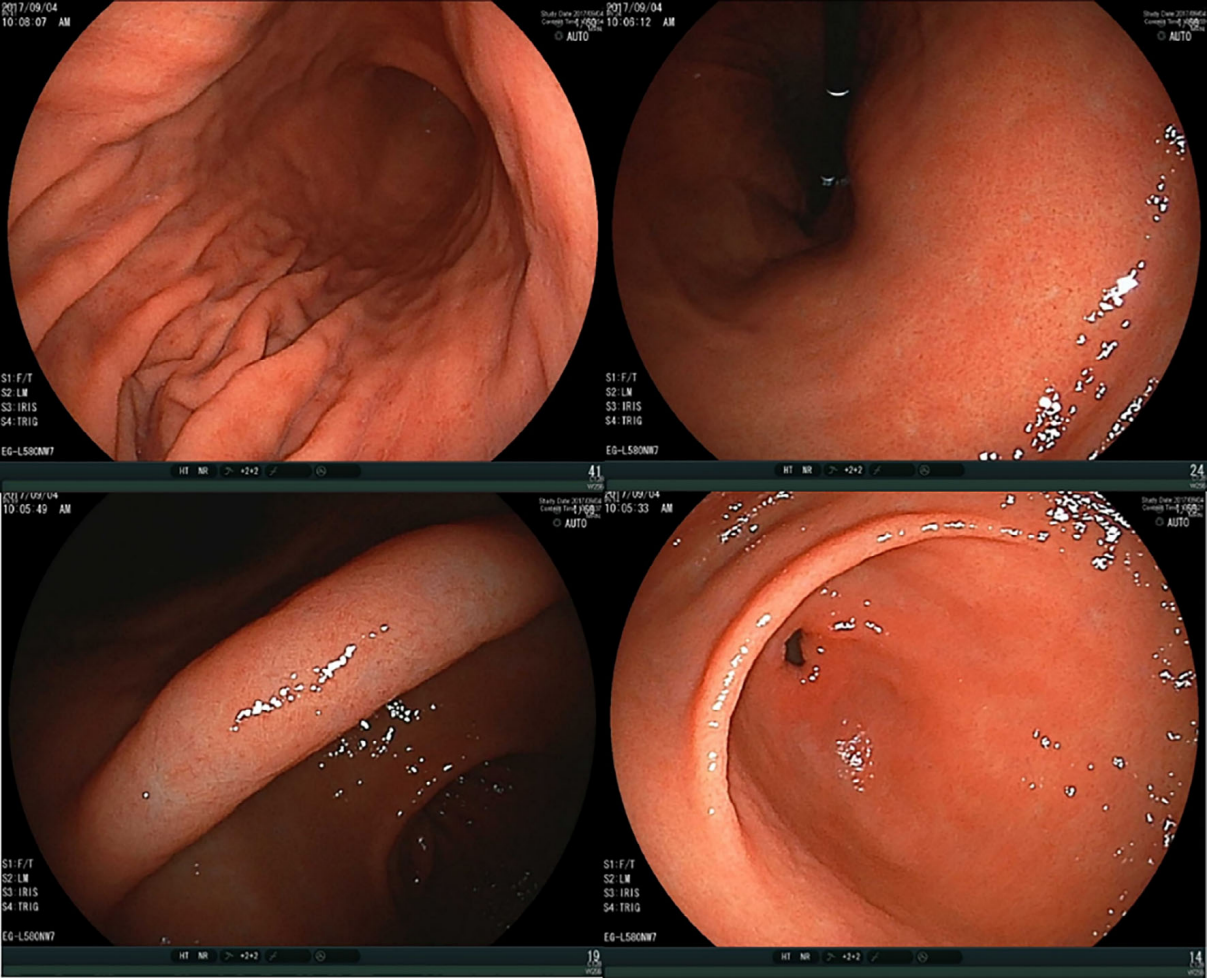
Endoscopic images of a false‐negative case of the endoscopic judgment for *Helicobacter pylori* infection. Smooth gastric mucosa without apparent atrophy is seen. The ABC method was Group B (*H. pylori* antibody titer >13, PGI 74.3, PGII 17.4, and PGI/II 4.3), and current infection of *H. pylori* was confirmed by histological findings of neutrophil infiltration in the gastric mucosa. PG, pepsinogen

Regarding factors associated with the false‐negative endoscopic judgment for *H. pylori* infection, the ABC classification and titers of *H. pylori* antibody of the 52 cases are presented in Table 4. Fifty of them were Group B of the ABC method, supposed to have gastritis with mild mucosal atrophy. In 39 of the 50 cases, the antibody titers were 3 ~ <10, a weak‐positive range, leaving the possibility that endoscopic judgment of nongastritis was correct in these cases. However, clinically significant elevation of the antibody to 10 or more, suggesting current *H. pylori* infection, was present in the other 11 cases. In the multivariate logistic regression analysis, weakly positive antibody titers (odds ratio = 15.7, 95% CI: 8.45‐29.0, *P* < .01) and negative PG test (5.45, 2.14‐13.9, *P* < .01) were identified as independent factors associated with the false‐negative endoscopic judgment (Table 5). Typical endoscopic images of one such case is presented in Figure 2, showing no apparent gastric atrophy or signs of gastritis indicating *H. pylori* infection. In this case, current infection of *H. pylori* was confirmed by histological findings of neutrophil infiltration in the gastric mucosa, that is, active gastritis. These findings indicate that making the diagnosis of *H. pylori* by endoscopic judgment is sometimes difficult, even with current *H. pylori* infection when gastric atrophy has not progressed significantly.

**TABLE 4:** The ABC classification and the titer of *Helicobacter pylori* antibody in the false‐negative cases of endoscopic evaluation for *H. pylori* infection

ABC classification	n	Titer of *H. pylori* antibody^a^		
		<3	3 ~ <10	≥10
Group B	50		39	11
Group C	1			1
Group D	1	1		

^a^ ≥3 U/mL, a cut‐off value recommended for the setting of gastric cancer screening program; ≥10 U/mL, an original cut‐off value for clinical practice.

**TABLE 5:** Multivariate logistic regression analysis of factors associated with false‐negative results of the endoscopic judgment for *Helicobacter pylori* infection

	Odd ratio	95% CI	*P* value
Male	0.72	0.40‐1.30	.28
Age (>60)	0.54	0.29‐1.02	.06
Use of digestive medicine^a^	0.92	0.35‐2.38	.86
Titer of *H. pylori* antibody (<10)	15.7	8.45‐29.0	<.01
Negative PG test	5.45	2.14‐13.9	<.01

*Note*: PG test, pepsinogen test; positive if PGI ≤70 ng/mL and PG I/II ratio ≤3.0.

Abbreviation: CI, confidence interval.

^a^ H2‐receptor antagonist, proton pump inhibitor, potassium‐competitive acid blocker, and gastric mucosa–protective drugs.

**FIGURE 3:**
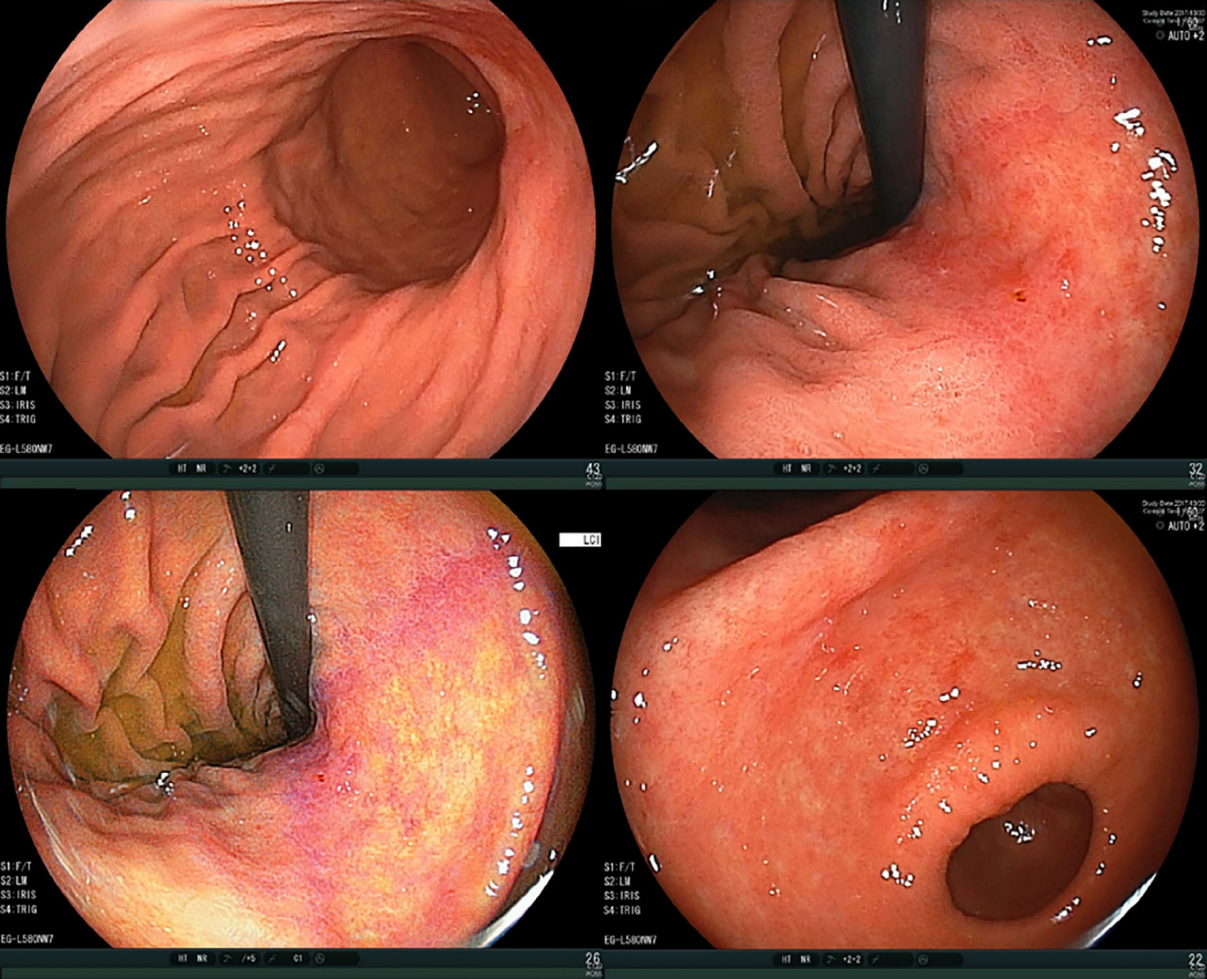
Endoscopic images of a false‐negative case of the ABC method for *Helicobacter pylori* infection (past infection). Typical map‐like redness is apparent in the lesser curvature of the gastric body, indicating past infection, but the ABC method was Group A (antibody titer <3, PGI 29.7, PGII 6.2, and PGI/II 4.8). Because subjects who had a history of eradication therapy were excluded in this study, natural or accidental eradication of *H. pylori* was likely

Regarding the false‐negative results of the ABC method for *H. pylori* infection, apparent active gastritis, that is, current *H. pylori* infection, was present in three cases (7.3%). Most cases (38/41, 92.7%) were judged to have inactive gastritis endoscopically, suggesting past *H. pylori* infection; because we excluded subjects who had a history of eradication therapy, *H. pylori* in these subjects was likely to have been eradicated naturally or accidentally. A typical case is presented in Figure 3.

## 4 DISCUSSION

In this prospective study, we tried to address the major drawback of the population‐based gastric cancer screening program in Japan, that is, screening without consideration of *H. pylori* infection status. To try to overcome this drawback, we used endoscopic judgment of *H. pylori* infection status according to the Kyoto classification of gastritis. However, we found that the false‐negative rate for *H. pylori* infection was 16.3% when the serum test (the ABC method) was used as a reference standard (11% after re‐evaluation by experienced endoscopists, but still a disquieting rate). In a recent work reported by Yoshii et al,^12^ the sensitivity for past or current infection by endoscopic judgment using the Kyoto classification of gastritis was about 70‐80% when eradication history and serum *H. pylori* antibody titer were used as a reference standard. Thus, use of the endoscopic assessment of *H. pylori* infection status left challenges.

We believe, nonetheless, that the Kyoto classification of gastritis is a well‐designed and sophisticated way for judging *H. pylori* infection based on endoscopic findings. In the present study, it was indeed easy to diagnose *H. pylori* infection, regardless of current or past infection, in most typical cases, although atypical cases sometimes had endoscopic features like those of stomach never infected with the bacterium. The false‐negative endoscopic judgment for *H. pylori* infection occurred mostly in cases without significant gastric mucosal atrophy. In the natural history of *H. pylori* infection, the bacterium first infects in the gastric antrum, and the resulting gastritis and mucosal atrophy gradually progress to the gastric body. In the Kyoto classification of gastritis, evaluation of gastric atrophy is important; when gastric mucosal atrophy is endoscopically negligible, nongastritis, meaning no history of *H. pylori* infection, is usually the diagnosis unless mucosal redness or swelling, that is, features of current infection, are observed. It is not difficult to judge gastric mucosal atrophy endoscopically by identifying the atrophic border described by Kimura and Takemoto,^16^ but recognizing redness and swelling of the gastric mucosa often depends on subjective judgment. Thus, in cases without significant gastric mucosal atrophy, the endoscopic diagnosis of *H. pylori* infection can be difficult. Simultaneous evaluation of serum *H. pylori* antibody may be helpful to avoid such false‐negative results. In addition, it is also important to continually educate endoscopists participating in gastric cancer screening programs about the Kyoto classification of gastritis, evidenced by the false‐negative rate of endoscopic diagnosis decreasing from 16% to 11% after re‐evaluation by experienced endoscopists.

On the other hand, false‐negative results of the serum‐based ABC method occurred in 19.0% with the endoscopic diagnosis as a reference standard; the value decreased to 9.2% after re‐evaluation of endoscopic findings, but nearly 10% still seems a high value. The endoscopic diagnosis of the false‐negative cases by the ABC methods was mostly inactive gastritis, that is, past infection of *H. pylori*. We excluded subjects with a history of eradication therapy. Thus, it appears that natural or accidental eradication of *H. pylori* occurred in a certain proportion, and that the ABC method can be negative in such cases. To increase the sensitivity of the ABC method, the arranged cut‐off values of PGs were recently reported^21^; whether this arrangement will be useful in the gastric cancer screening program needs investigation.

Limitations of our study are these: (a) The diagnosis of *H. pylori* infection by the endoscopic method or the ABC method was useful, but the sensitivity by either method was not ideal. In determining *H. pylori* status, we used the ABC method as a reference standard for evaluation of the endoscopic diagnosis and endoscopic diagnosis as a reference standard for comparison with the ABC method: the dependability of both methods, however, appears limited. Other methods for determining *H. pylori* infection status, for example, bacterial culture, histological evaluation of gastritis, stool antigen test, and urease‐based tests, are more reliable. However, gastric biopsy for this purpose is not recommended in population‐based screening programs, and urease‐based tests or stool antigen quantification do not detect past infection. Thus, we arbitrarily calculated the false‐negative rates by using endoscopic diagnosis or the result of the ABC method as a reference standard. (b) Endoscopists with various levels of skill and knowledge for endoscopic diagnosis according to the Kyoto classification of gastritis, that is, from senior resident to board‐certified specialists performed EGD, but doctors in local private clinics were not included. Application of our findings to population‐based gastric cancer screening programs in private clinics needs to be tested.

In conclusion, endoscopic evaluation of gastritis according to the Kyoto classification is useful for judging *H. pylori* infection status in population‐based gastric cancer screening, but false‐negative results for *H. pylori* infection may occur, especially in the presence of mild gastric atrophy. To avoid this, we recommend adding *H. pylori* antibody testing in endoscopic gastric cancer screening. *H. pylori*–infected persons identified through this approach could be offered eradication therapy for current infection, and those with past infection could be carefully surveilled.

## FUNDING

None.

## CONFLICT OF INTEREST

There is no conflict of interest in this study. The work has not been published previously.

## AUTHOR CONTRIBUTIONS

Conceptualization: Mami Hirai, Yuichi Shimodate, and Motowo Mizuno

Project Administration: Mami Hirai and Yuichi Shimodate

Formal Analysis: Ryosuke Hirai

Visualization: Mariko Minami, Sho Ishikawa, Takafumi Kanadani, Rio Takezawa, Akira Doi, Naoyuki Nishimura, Hirokazu Mouri, and Kazuhiro Matsueda, Hiroshi Yamamoto

Supervision: Motowo Mizuno

Writing‐Review and Editing: Motowo Mizuno

Writing‐Original Draft: Ryosuke Hirai

All authors have read and approved the final version of the manuscript

The corresponding author had full access to all of the data in this study and takes complete responsibility for the integrity of the data and the accuracy of the data analysis.

## TRANSPARENCY STATEMENT

The lead author affirms that this manuscript is an honest, accurate, and transparent account of the study being reported; that no important aspects of the study have been omitted; and that any discrepancies from the study as planned (and, if relevant, registered) have been explained.

## Supporting information


**Table S1.** Characteristics of study subjects by the results of the ABC method

## Data Availability

All relevant data and materials are provided within the manuscript.
