# Supplementary material for: Feasibility of endoscopic evaluation of Helicobacter pylori infection status by using the Kyoto classification of gastritis in the population‐based gastric cancer screening program: A prospective cohort study
Source: Health Sci Rep. 2021 Jul 14;4(3):e325. doi: 10.1002/hsr2.325 (PMC8279217; doi:10.1002/hsr2.325)
Supplement: Supplementary file 1 — Table S1. Characteristics of study subjects by the results of the ABC method [file HSR2-4-e325-s001.docx]

Supplementary Table S1. Characteristics of study subjects by the results of the ABC method

| ABC method |  | A | B-D |
| --- | --- | --- | --- |
| N |  | 1,312 | 472 |
| Age (years) |  | 53.0 (±11.4) | 58 (±12.5) |
| Male |  | 661 (50.1%) | 256 (54.2%) |
| Use of digestive medicine |  | 134 (10.2%) | 47 (10.0%) |
| Histamine H2-receptor antagonist |  | 13 (1.0%) | 5 (1.1%) |
| Proton pump inhibitor |  | 71 (5.4%) | 17 (3.6%) |
| Potassium-competitive acid blocker |  | 8 (0.6%) | 2 (0.4%) |
| Gastric neoplasms | Cancer | 0 (0%) | 7 (1.5%) |
|  | Adenoma | 0 (0%) | 2 (0.4%) |
| Kyoto classification of gastritis |  |  |  |
| Atrophy |  | 89 (6.8%) | 381 (80.7%) |
| Intestinal metaplasia |  | 23 (17.5%) | 188 (39.8%) |
| Diffuse and/or spotty redness |  | 13 (1.0%) | 266 (56.4%) |
| Mucosal swelling and/or enlarged fold |  | 35 (2.7%) | 280 (59.3%) |
| Nodularity |  | 3 (0.2%) | 18 (3.8%) |
| RAC on angular region or antrum |  | 1,223 (93.2%) | 102 (21.6%) |
| Endoscopic diagnosis | Non-gastritis | 1,215 (92.6%) | 78 (16.5%) |
|  | Active or inactive | 89 (6.8%) | 381 (80.7%) |
|  | Undefined | 8 (0.6%) | 15 (3.2%) |

RAC; regular arrangement of collecting venules.
